# Tubercular Mortality

**Published:** 1914-11

**Authors:** 


### Tubercular Mortality. Have We Reached the Limit?

The Buffalo Sanitary Bulletin gives the actual and proportionate death rates from tuberculosis for Buffalo, from 1882 to 1913. With some fluctuations which are in some instances readily explained by inability to estimate accurately the population for any given year, it mey be stated that the mortality ranged close to 2 per thousand population until 1892, that it then fell pretty steadily till 1900 when it was 1.2 and that it has remained nearly at this point ever since. In comparing this statement with the figures originally given, it should be borne in mind that we have allowed for inability to check the population accurately between census years. Quite aside from the impossibility of estimating the population accurately, fluctuations of any death rate from year to year have no great significance. It is easy to understand why the first attempts, with scientific knowledge and increased executive power, should produce marked results and why the problem should then become increasingly difficult. But it is somewhat discouraging that the mortality should remain practically stationary for nearly 15 years, especially when we are speaking in terms of aggregate mortality and not of the proportion of all deaths due to a particular cause. We have at other times published other statistics of tuberculosis which tend to show that this disease is diminishing in mortality at least as rapidly as the general death rate. Are there peculiar local factors applying to Buffalo? Off hand, we should say that the only possible unfavorable factors of this sort, applicable to tuberculosis, are climatic, and neither hygienic, sanitary nor nutritive—of course, in a relative sense, with due reference to the conditions of other cities. Or, have we reached nearly the minimum death rate from tuberculosis under conditions of life at present securable?

